# 

**Published:** 1861-06

**Authors:** 


☞ Central Ohio Dental Association.—The second semi-annual meeting of this new society will be held in Newark, Ohio, on the 10th and 11th of July, 1861. All dentists in Central Ohio feeling an interest in the advancement and elevation of the profession, are earnestly requested to be present, and it is hoped that each one will be prepared to open his budget, that all may contribute to the general fund of knowledge, and learn lessons of wisdom from each other's experience. Thomas M'Cune, *Cor. Sec.*

### DENTAL REGISTER,

OF THE WEST.

**Issued Monthly, at $3 a year in advance.**

DEVOTED TO THE INTERESTS OF THE DENTAL PROFESSION.

**Edited by J. TAFT and GEO. WATT.**

The Volume commences in January and closes in December.

The Register being issued monthly is always fresh, therefore indispensable to the practitioner who desires to keep posted up with the new inventions and improvements which are daily developed. Neither expense nor effort will be spared to give value and variety to its contents. Articles will be illustrated with well executed engravings, whenever they will add to the interest or assist in explanation.

Its extensive circulation and frequency of issue makes it the BEST DENTAL ADVERTISING MEDIUM in the United States.

☞ CONTRIBUTIONS SOLICITED.

Address Original Communications to **Geo. Watt, Xenia, O.**

Exchanges to **J. Taft**, or **Dental Register, Cincinnati, O.**

Business Gommunications, to

**JNO. T. TOLAND, Publisher and Proprietor,**
38 West Fourth Street, Cincinnati, O.

Communications must reach the Editor as early as the 15th, to insure insertion in the next issue.

